# The risk of developing type 2 diabetes mellitus among the students of Hail University, Saudi Arabia

**DOI:** 10.3389/fpubh.2023.1278103

**Published:** 2023-09-26

**Authors:** Bahia Glalal Abd El-Razik Siam, Shimaa Mohamed Abdou Rizk, Soha Kamel Mosbah Mahmoud

**Affiliations:** ^1^Medical-Surgical Nursing Department, College of Nursing, University of Hail, Hail, Saudi Arabia; ^2^Medical-Surgical Nursing Department, Faculty of Nursing, Mansoura University, Mansoura, Egypt; ^3^Community Health Nursing Department, College of Nursing, University of Hail, Hail, Saudi Arabia

**Keywords:** diabetes mellitus, risk, Saudi Arabia, university, students

## Abstract

**Background:**

Globally, it is estimated that approximately 537 million adults are living with diabetes. Of them, more than 90% have type 2 diabetes (T2DM). In 2023, a previous meta-analysis showed that the prevalence of T2DM among the general adult population in Saudi Arabia was 28%. This study was conducted to assess the risk of developing T2DM among the students at Hail University, Saudi Arabia.

**Methods:**

This cross-sectional study was conducted in 2022/2023 among a census sample of 740 students (both genders, aged 17–26 years) studying at nine colleges of Hail University, Saudi Arabia. The diabetes risk score was assessed using the Australian Type 2 Diabetes Risk Assessment Tool (AUSDRISK). Anthropometric measurements were measured and recorded using standard methods. Socio-demographic variables were also obtained with an interview-based questionnaire. Statistical analysis was performed using SPSS version 25.

**Results:**

A total of 740 students were included in the final analysis. Of them, 274 (37.0%) were male students and 466 (63.0%) were female students. The mean age of the study participants is 19.9 ± 1.6 years. The findings showed that 61.9% of the study participants were at intermediate and high risk of diabetes (59.7 and 2.2%, respectively). The majority 85.7% of male students were at intermediate risk of diabetes, and 5.8% were at high risk of diabetes. In total, 44.4% of female students were at intermediate risk of diabetes, and none of them were at high risk of diabetes. For the following variables (age, gender, college name, area of the university, academic years, weight, height, and BMI), the differences were statistically significant between different categories of diabetes risk scores (*P*-values < 0.005).

**Conclusion:**

More than half of the students at the Hail University of Saudi Arabia have an intermediate and high risk of T2DM. Male students are at a higher risk compared to female students. The high risk of T2DM among university students should be seriously considered.

## Introduction

Globally, it is estimated that approximately 537 million adults are living with diabetes mellitus (DM), i.e., approximately 10.5% of the global population. Of them, more than 90% have type 2 diabetes (T2DM) (1). The Eastern Mediterranean region has the second-highest prevalence of DM worldwidde, and approximately 25% of people in this area have DM (2). In 2023, a previous meta-analysis showed that the prevalence of T2DM among the general adult population in Saudi Arabia was 28% (3). According to the International Diabetes Federation (IDF), in 2019, approximately 18.3% of Saudi adults have DM, which is a worrying estimate, and over 50% of Saudi adults will have DM by 2030 (4). T2DM is a significant public health concern in Hail City. It is still a terrible, chronic illness, despite the amazing advances in clinical research and diabetes science. Furthermore, T2DM is rapidly increasing in prevalence among people of all ages and both genders in Hail City (5).

One or more T2DM risk factors may emerge throughout the university years due to the significant socio-behavioral health changes that are linked with this age group. The rising prevalence in the younger age group has been linked to lifestyle factors such as unhealthy eating habits and insufficient exercise, stress from exams and ongoing evaluation, smoking, and drinking, which are common among college-age groups, and non-modifiable risk factors, which include a family history of DM (6).

Being overweight and/or obese relates to unhealthy lifestyle choices that can lead to impaired glucose metabolism, hypertension, and dyslipidemia, all of which increase a person's chance of developing T2DM in the future (7). Furthermore, one of the primary factors in the pathophysiology of T2DM is insulin resistance, which is caused by an increase in body weight. Previous studies indicated that T2DM can be prevented and treated very well by losing body weight (8, 9).

Identifying the T2DM risk factors among university students is essential for effective promotion, prevention, and intervention programs. However, it is still unclear whether a gradual increase in the number of at-target risk factors directly correlates with patient outcomes. A previous study revealed that the increase in the number of risk factors at target (also, laboratory findings) correlates with better cardiovascular-free survival in T2DM patients (10). To the best of our knowledge, no previous studies have focused on college students and their risk for T2DM in Hail City, Saudi Arabia. Despite the fact that many of the students engage in high-risk lifestyle habits, better identifying and comprehending metabolic dysregulation in high-risk individuals is a critical public health issue as T2DM prevalence among college-age groups rises. Therefore, the current study was conducted to assess the risk of developing T2DM among the students of Hail University, Saudi Arabia.

## Materials and methods

### Study design, period, and setting

This cross-sectional study was conducted in 2022/2023 among university students studying at nine colleges of Hail University, Saudi Arabia.

### Study participants and sampling method

In this study, 812 students were invited to participate, of whom 72 were excluded. Then, a total of 740 students (both genders, aged 17–26 years, from all academic levels, and from all colleges) who were studying at nine colleges of Hail University, Saudi Arabia, were included in the current study. The students were selected using a census sampling method. Pregnant or lactating female students were excluded from the study to avoid any possible bias as pregnancy and lactation changed a woman's weight and nutritional status, and some of them were on vacation from the university. In addition, students with any type of serious illness, such as cancer or acute myocardial infarction, were also excluded from the study as they were unable to participate due to their acute illness.

### Data collection

#### An interview-based questionnaire

An interview-based questionnaire was employed to assess the socio-demographic variables of the studied students, including age (years), residence, nationality, college name, area of the university, and academic years (levels).

#### Assessment of anthropometric measurements

Height (cm) and weight (kg) were measured and recorded using standard methods (11). In addition, the body mass index (BMI) was calculated by dividing weight in kilograms by the square of height in meters.

#### Assessment of the diabetes risk score

The diabetes risk score was assessed using the Australian Type 2 Diabetes Risk Assessment Tool (AUSDRISK), adopted from the “Baker Heart and Diabetes Institute” (12). The tool consists of 10 questions as risk factors for developing T2DM. The answers to the items in the AUSDRISK tool ranged from 0 to 7. The total diabetes risk score was calculated and then categorized as follows: (1) low diabetes risk (if the total scores are 5 or less); (2) intermediate diabetes risk (if the total scores are 6–11); and (3) high diabetes risk (if the total scores are 12 and more). In the current study, the questionnaire was translated and administered in the Arabic language. The questionnaire language appropriateness, content validity, question comprehensibility, and refining were achieved by five experts from relevant fields before actual distribution among the students. The reliability, consistency, and stability of the questionnaire were tested using Cronbach's alpha coefficient (α = 0.87).

### Pilot study

Before the data collection process, to ensure the survey's acceptance and consistency, a pilot study was undertaken among 30 students. After that, small adjustments were made considering the pilot study's findings.

### Statistical analysis

Statistical analysis was performed using SPSS version 25. Data are expressed as means ± SD for continuous variables and as percentages for categorical variables. The differences between means were tested by using an independent sample *t-*test and a one-way ANOVA. The chi-square test was used to examine differences in the prevalence of different categorical variables. A *P*-value < 0.05 was considered statistically significant.

## Results

A total of 740 students were included in the final analysis. Of them, 274 (37.0%) were male students and 466 (63.0%) were female students. The mean age of the study participants is 19.9 ± 1.6 years. In total, 47.0% of participants were 17 to <20 years of age (urban 81.2%), the majority (98.9%) were Saudi, and 24.5% were from the preparatory college. In addition, 72.8% were selected from the women's building, and 44.9% were in their first-year academic level. Regarding BMI (kg/m^2^), the results demonstrated that 13.1% of the students were underweight, 23.6% were overweight, and 11.9% were obese. For the following variables (age, college name, area of the university, academic years, weight, height, and BMI), the differences were statistically significant between male and female students (*P*-values < 0.005 for all) (Table 1).

**Table 1:** Characteristics of the study participants.

**Variables**	**Total, *n* (%) 740 (100)**	**Male students, *n* (%) 274 (37.0)**	**Female students, *n* (%) 466 (63.0)**	***P*-value**
**Age (years) Mean** ±**SD: 19.9** ±**1.6**				
17 to < 20	348 (47.0)	188 (54.0)	160 (46.0)	0.001
20 to < 23	247 (33.4)	61 (24.7)	186 (75.3)	
23 to 26 years	145 (19.6)	25 (17.2)	120 (82.8)	
**Residence**				
Rural	139 (18.8)	59 (42.4)	80 (57.6)	0.145
Urban	601 (81.2)	215 (35.8)	386 (64.2)	
**Nationality**				
Saudi	732 (98.9)	271 (37.0)	461 (63.0)	0.620
Non-Saudi	8.0 (1.1)	3.0 (37.5)	5.0 (62.5)	
**College name**				
Nursing	166 (22.4)	5.0 (3.0)	161 (97.0)	0.001
Engineering	15 (2.0)	2.0 (13.3)	13 (86.7)	
Sciences	171 (23.1)	102 (59.6)	69 (40.4)	
Computer sciences	17 (2.3)	3.0 (17.6)	14 (82.4)	
Preparatory	181 (24.5)	140 (77.3)	41 (22.7)	
Education	27 (3.6)	4.0 (14.8)	23 (85.2)	
Informatics	21 (2.8)	1.0 (4.8)	20 (95.2)	
Medical sciences	26 (3.5)	17 (65.4)	9.0 (34.6)	
Arts	116 (15.7)	0.0 (0.0)	116 (100)	
**Area of the university**				
Women's building	539 (72.8)	99 (18.4)	440 (81.6)	0.001
Men's building	175 (23.6)	171 (97.7)	4.0 (2.3)	
Other branches (includes female and male students)	26 (3.5)	4.0 (15.4)	22 (84.6)	
**Academic years (levels)**				
First year	332 (44.9)	133 (40.1)	199 (59.9)	0.001
Second year	205 (27.7)	117 (57.1)	88 (42.9)	
Third year	102 (13.8)	9.0 (8.8)	93 (91.2)	
Fourth year	78 (10.5)	15 (19.2)	63 (80.8)	
Fifth year	23 (3.1)	0.0 (0.0)	23 (100)	
**Body mass index (kg/m** ^2^ **)**				
Underweight (< 18.5)	97 (13.1)	27 (27.8)	70 (72.2)	0.015
Normal weight (18.5–24.9)	380 (51.4)	133 (35.0)	247 (65.0)	
Overweight (25–29.9)	175 (23.6)	71 (40.6)	104 (59.4)	
Obesity (≥30)	88 (11.9)	43 (48.9)	45 (51.1)	
**Weight (kg): Mean** **±SD**	64.0 ± 16	73.1 ± 16	58.6 ± 13	0.001
**Height (m): Mean** **±SD**	1.63 ± 0.09	1.73 ± 0.06	1.58 ± 0.05	0.001
**BMI (kg/m**^**2**^**): Mean** **±SD**	23.7 ± 5.3	24.4 ± 5.5	23.3 ± 5.3	0.007

Data are expressed as means ± SD for continuous variables and as percentages for categorical variables. The differences between means were tested using an independent sample *t*-test. The chi-square test was used to examine differences in the prevalence of different categorical variables. A *P*-value < 0.05 was considered statistically significant. SD, standard deviation.

As shown in Table 2, the findings revealed that all of the study participants were under 35 years old, 63.0% were female students, 100% had Asian ethnicity, 54.6% had a first-degree history of diabetes, only 1.4% had hyperglycemia, 1.2% used medications for high blood pressure, and 8.4% were smokers. In addition, 34.7% did not practice regular physical activity for at least 2.5 h per week, and 47.7% had a high waist circumference (90–100 cm for male students and 80–90 cm for female students). The overall mean diabetes risk score of the study participants was 6.35 ± 2.56 (8.31 ± 2.34 in male students vs. 5.20 ± 1.91 in female students). Concerning the categories of diabetes risk score, the results showed that 38.1% of the study participants were at low risk of diabetes, 59.7% were at intermediate risk, and only 2.2% were at high risk of diabetes. For the following variables (gender score, first-degree family history of diabetes, current history of hyperglycemia, current daily use of tobacco products such as cigarettes, waist measurement risks for Asians, and the categories of diabetes risk score), the differences were statistically significant between male and female students (*P-*values < 0.001 for all) (Table 2).

**Table 2:** The diabetes risk scores of the study participants by gender and based on the Australian type 2 diabetes risk assessment tool (AUSDRISK).

**Variables**	**Total, *n* (%) 740 (100)**	**Male students, *n* (%) 274 (37.0)**	**Female students, *n* (%) 466 (63.0)**	***P*-value**
**Age group**				
Under 35 (0 points)	740 (100)	274 (37.0)	466 (63.0)	-
**Gender**				
Females (0 points)	466 (63.0)	0.0 (0.0)	466 (100)	0.001
Males (3 points)	274 (37.0)	274 (100)	0.0 (0.0)	
**Ethnicity**				
Asians (2 points)	740 (100)	274 (37.0)	466 (63.0)	-
Others (0 points)	0.0 (0.0)	0.0 (0.0)	0.0 (0.0)	
**First-degree family history of diabetes**				
Positive (3 points)	404 (54.6)	172 (42.6)	232 (57.4)	0.001
Negative (0 points)	336 (45.4)	102 (30.4)	234 (69.6)	
**Current history of hyperglycemia (e.g., in a health examination, during an illness, or during pregnancy)**				
No (0 points)	730 (98.6)	264 (36.2)	466 (63.8)	0.001
Yes (6 points)	10 (1.4)	10 (100)	0.0 (0.0)	
**Currently using high blood pressure medication**				
No (0 points)	731 (98.8)	272 (37.2)	459 (62.8)	0.290
Yes (2 points)	9.0 (1.2)	2.0 (22.2)	7.0 (77.8)	
**Current daily use of tobacco products, such as cigarettes**				
No (0 point)	678 (91.6)	219 (32.3)	459 (67.7)	0.001
Yes (2 points)	62 (8.4)	55 (88.7)	7.0 (11.3)	
**Frequency of eating vegetables or fruits**				
Every day (0 points)	165 (22.3)	61 (37.0)	104 (63.0)	0.531
Not every day (1 point)	575 (77.7)	213 (37.0)	362 (63.0)	
**Practicing regular physical activity for at least 2.5 h per week (e.g., 30 min a day on 5 or more days a week)**				
Yes (0 points)	432 (58.4)	167 (38.7)	265 (61.3)	0.156
No (2 points)	308 (41.6)	107 (34.7)	201 (65.3)	
**Waist measurement (cm) risks for Asian**				
For men: 90 cm or less; for women: 80 cm or less (0 points)	202 (27.3)	136 (67.3)	66 (32.7)	0.001
For men: 90–100 cm; for women: 80–90 cm (4 points)	353 (47.7)	115 (32.6)	238 (67.4)	
For men: 100 cm or more; for women: 90 cm or more (7 points)	185 (25.0)	23 (12.4)	162 (87.6)	
**Total risk score (Mean** **±SD)**	6.35 ± 2.56	8.31 ± 2.34	5.20 ± 1.91	0.001
**Low risk**	282 (38.1)	23 (8.2)	259 (91.8)	0.001
**Intermediate risk**	442 (59.7)	235 (53.2)	207 (46.8)	
**High risk**	16 (2.2)	16 (100)	0.0 (0.0)	

Data are expressed as means ± SD for continuous variables and as percentages for categorical variables. The differences between means were tested using an independent sample *t*-test and a one-way ANOVA. The chi-square test was used to examine differences in the prevalence of different categorical variables. A *P*-value < 0.05 was considered statistically significant. SD, standard deviation; low diabetes risk (if the total score is 5 or less); intermediate diabetes risk (if the total score is 6 to 11); and high diabetes risk (if the total score is 12 or more).

**Figure 1:**
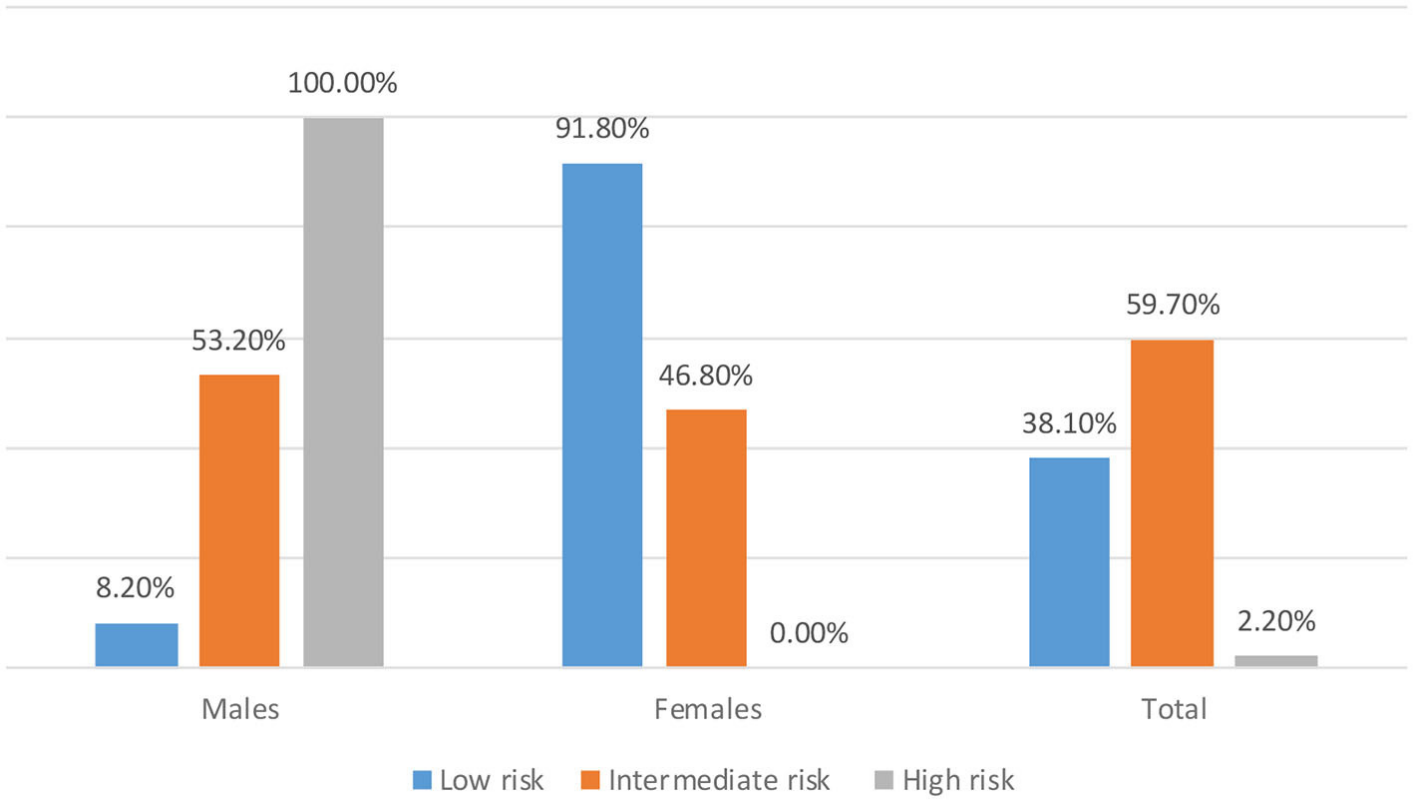
Distribution of the study participants based on the categories of diabetes risk score (AUSDRISK) by gender.

Figure 1 shows the distribution of the study participants based on the categories of diabetes risk score (AUSDRISK) by gender. The main results demonstrated that 61.9% of the study participants were at intermediate and high risk of diabetes (59.7 and 2.2%, respectively). In addition, 85.7% of male students were at intermediate risk of diabetes, 5.8% were at high risk of diabetes, and only 8.5% of them were at low risk of diabetes. While 44.4% of female students were at intermediate risk of diabetes, none of them were at high risk of diabetes (their AUSDRISK total score was <12), and more than half of them (55.6%) were at low risk of diabetes.

The results also demonstrated that 63.4% of students aged 23–26 years were at intermediate risk of diabetes, and 4.0% of students aged 17 to <20 were at high risk of diabetes. In addition, the majority (85.8%) of male participants were at intermediate risk of diabetes, while 5.8% were at high risk of diabetes. In total, 64.0% of rural participants were at intermediate risk of diabetes, while 58.7% of urban participants were at intermediate risk of diabetes. Furthermore, 60.0% of the Saudi participants were at intermediate risk of diabetes, and 2.2% were at high risk of diabetes. In total, 72.4% of the students in the preparatory college were at intermediate risk of diabetes, and 5.5% were at high risk of diabetes. A large percentage of the students (86.3%) in the men's building of the university were at intermediate risk of diabetes, and 4.6% were at high risk of diabetes. The highest percentage (67.9%) of intermediate diabetes risk was found among the students in the fourth academic year, while the highest percentage (3.0%) of high risk of diabetes was found among the students in the first academic year. Furthermore, 75.0 and 4.5% of the students with obesity had an intermediate and high risk of diabetes, respectively. Moreover, the mean BMI (kg/m^2^) for students with intermediate and high risk of diabetes were 24.31 ± 5.5 and 25.67 ± 6.2, respectively. Additionally, for the following variables (age, gender, college name, area of the university, academic years, weight, height, and BMI), the differences were statistically significant between different categories of diabetes risk scores (*P*-values < 0.005 for all) (Table 3).

**Table 3:** Relationship between the characteristics of the study participants and the categories of diabetes risk scores.

**Variables**	**Low risk, *n* (%) 282 (38.1)**	**Intermediate risk, *n* (%) 442 (59.7)**	**High risk, *n* (%) 16 (2.2)**	***P*-value**
**Age (years)**				
17 to < 20	121 (34.8)	213 (61.2)	14 (4.0)	0.004
20 to < 23	108 (43.7)	137 (55.5)	2.0 (0.8)	
23–26 years	53 (36.6)	92 (63.4)	0.0 (0.0)	
**Gender**				
Males	23 (8.4)	235 (85.8)	16 (5.8)	0.001
Females	259 (55.6)	207 (44.4)	0.0 (0.0)	
**Residence**				
Rural	46 (33.1)	89 (64.0)	4.0 (2.9)	0.356
Urban	236 (39.3)	353 (58.7)	12 (2.0)	
**Nationality**				
Saudi	277 (37.8)	439 (60.0)	16 (2.2)	0.349
Non-Saudi	5.0 (62.5)	3.0 (37.5)	0.0 (0.0)	
**College name**				
Nursing	95 (57.2)	71 (42.8)	0.0 (0.0)	0.001
Engineering	8.0 (53.3)	7.0 (46.7)	0.0 (0.0)	
Sciences	45 (26.3)	120 (70.2)	6.0 (3.5)	
Computer sciences	11 (64.7)	6.0 (35.3)	0.0 (0.0)	
Preparatory	40 (22.1)	131 (72.4)	10 (5.5)	
Education	11 (40.7)	16 (59.3)	0.0 (0.0)	
Informatics	9.0 (42.9)	12 (57.1)	0.0 (0.0)	
Medical sciences	2.0 (7.7)	24 (92.3)	0.0 (0.0)	
Arts	61 (52.6)	55 (47.4)	0.0 (0.0)	
**Area of the university**				
Women's building	254 (47.1)	277 (51.4)	8.0 (1.5)	0.001
Men's building	16 (9.1)	151 (86.3)	8.0 (4.6)	
Other branches	12 (46.2)	14 (53.8)	0.0 (0.0)	
**Academic years (levels)**				
First year	135 (40.7)	187 (56.3)	10 (3.0)	0.037
Second year	64 (31.2)	135 (65.9)	6.0 (2.9)	
Third year	49 (48.0)	53 (52.0)	0.0 (0.0)	
Fourth year	25 (32.1)	53 (67.9)	0.0 (0.0)	
Fifth year	9.0 (39.1)	14 (60.9)	0.0 (0.0)	
**Body mass index (kg/m** ^2^ **)**				
Underweight (< 18.5)	47 (48.5)	50 (51.5)	0.0 (0.0)	0.003
Normal weight (18.5–24.9)	154 (40.5)	218 (57.4)	8.0 (2.1)	
Overweight (25–29.9)	63 (36.0)	108 (61.7)	4.0 (2.3)	
Obesity (≥30)	18 (20.5)	66 (75.0)	4.0 (4.5)	
**Weight (kg): Mean** **±SD**	58.07 ± 13.6	67.28 ± 16.5	78.8 ± 21.8	0.001
**Height (m): Mean** **±SD**	1.59 ± 0.06	1.66 ± 0.09	1.74 ± 0.05	0.001
**BMI (kg/m**^**2**^**): Mean** **±SD**	22.72 ± 4.8	24.31 ± 5.5	25.67 ± 6.2	0.001

Data are expressed as means ± SD for continuous variables and as percentages for categorical variables. The differences between means were tested using an independent sample *t*-test and a one-way ANOVA. The chi-square test was used to examine differences in the prevalence of different categorical variables. A *P*-value < 0.05 was considered statistically significant. SD, standard deviation; low diabetes risk (if the total score is 5 or less); intermediate diabetes risk (if the total score is 6 to 11); and high diabetes risk (if the total score is 12 or more).

## Discussion

T2DM is a chronic illness with a growing global incidence (1). The aim of this study was to assess the risk level for developing T2DM among students at Hail University, Saudi Arabia. The study results also show that less than half of the students who participated were in the age group from 17 to <20 years, with a mean age of 19.9 ± 1.6 years, while less than two-thirds of them were female students. This finding is supported by Sowndarya et al. (13) who found that 29.7% of participants were in the age group <30 years and 65.5% were female participants. Furthermore, the main results of the current study showed that more than half of the students at Hail University were at intermediate and high risk of diabetes (59.7 and 2.2%, respectively). This finding is in line with that of Gopalakrishnan et al. (14) who reported that 57.4% of the students were moderately at risk, and with other conclusions of the existing studies, which had reported that more people were at high risk of getting T2DM (15, 16). In addition, the findings of our study showed that male students were at a higher risk of diabetes compared to female students. This can be explained by the high levels of overweight and obesity and the low level of regular physical activity among male students compared to female students.

In the current study, and according to the AUSDRISK diabetes risk scores, the risk factors for diabetes among the studded students were distributed as follows: inadequate intake of fruits and vegetables, high waist circumference, first-degree family history of diabetes, inadequate physical activity, male gender, smoking, history of hyperglycemia, and use of high blood pressure medication, respectively. The findings demonstrated that all the studied students were younger than 35 years old, and nearly two-thirds of them were female students (items 1 and 2), indicating low-risk scores for developing DM. For ethnicity (item 3), all the studied students were Asian, which represented 2 scores. Our results reveal that a minority of the studied students were experiencing high blood glucose during the health examination, and a minority of them were taking antihypertensive medication (items 5 and 6). These findings are in line with those in a previous study (17), which showed that only 4.7% of participants took routine antihypertensive medicine, but 9.5% had elevated blood sugar levels. Moreover, the current study shows that more than half of the studied students had a positive first-degree family history of diabetes mellitus (item 4), more than one-third of them (35.5%) were overweight or obese, and more than two-fifths of them did not practice regular physical activity (item 9). This finding is consistent with Singh et al. (15), who found that DM risk components were a positive family history of diabetes, reduced physical exercise, and increased abdominal measurement. In addition, a previous study demonstrated that 5.1% of participants had diabetic parents, 9.5% had minimal or no physical activity, and 22.4% had a waist circumference >90 cm (18). The results of our study support these findings. As regards the frequency of eating vegetables and fruits (item 8), more than three-quarters of the studied students did not eat vegetables or fruits daily. This finding is consistent with a previous study (19), which showed that 78% of students consumed fewer fruits and vegetables, and the authors concluded that 59.73% of the students had an intermediate risk of diabetes based on the AUSDRISK. Concerning BMI, the present study reveals that more than one-third of the students were overweight or obese. The same findings were confirmed by Kes et al. (20), who reported that 23.2% of students were obese, while Sowndarya et al. (12) reported that the frequency of abdominal obesity was 44% in men and 84% in women.

Additionally, the current study reveals that there were highly statistically significant differences in the categories of diabetes risk scores of the studied students as regards their age, gender, college name, area of the university, academic years, weight, height, and BMI. This finding is congruent with that of Gopalakrishnan et al. (13) who stated that there is a significant correlation between risk variables such as the individual's age, gender, overweight/obesity, family history, and physical activity with the diabetes risk score. Letassy et al. (21) also showed that with increasing age, the number of diabetes risk factors increased. Additionally, none of the female students were at high risk of diabetes as their AUSDRISK total score was <12. In the current study, the low AUSDRISK total score among female students was due to their gender, low use of high blood pressure medication or smoking, high consumption of vegetables or fruits, high level of regular physical activity, and low weight and BMI. Further future studies are recommended to confirm these findings.

### Strengths and limitations

The main strength of the current study was its being the first study, which shows the risk of developing T2DM among the students of Hail University, Saudi Arabia, and its large sample size. The main limitations of this study are its cross-sectional design; the causal relationship could not be determined, and it limits the generalizability of our results. Unfortunately, we did not assess the dietary intake of this population. Having one determination of BMI could be extremely variable over time and can be misleading in the classification. Moreover, the lack of laboratory findings is another limitation.

## Conclusion

More than half of the students at the Hail University of Saudi Arabia have intermediate and high risks of T2DM. Male students are at a higher risk compared to female students. The high risk of T2DM among university students should be seriously considered, and policymakers should take steps to reduce it. Decision-makers should adopt clear health policies and interventions, including health promotion and education programs, to reduce major risk factors for diabetes among vulnerable youth, including university students.

## Data availability statement

The raw data supporting the conclusions of this article will be made available by the authors, without undue reservation.

## Ethics statement

The study protocol was approved by the Research Ethics Committee at Hail University (No.: H-2022-388). The studies were conducted in accordance with the local legislation and institutional requirements. The participants provided their written informed consent to participate in this study.

## Author contributions

BA: Conceptualization, Methodology, Supervision, Validation, Writing—review and editing. SA: Data curation, Investigation, Methodology, Writing—original draft. SM: Data curation, Formal analysis, Investigation, Methodology, Validation, Writing—original draft.
